# Multi-omics integration reveals TIM-4 as a master regulatory hub of neuroinflammation and neuronal cell death

**DOI:** 10.3389/fcell.2026.1876873

**Published:** 2026-08-21

**Authors:** Liang Chen, Yan-Yan Li, Li Han, Shiqi Lu

**Affiliations:** 1 Department of Emergency, the First Affiliated Hospital of Soochow University, Suzhou, Jiangsu, China; 2 Department of Emergency, Minhang Hospital, Fudan University, Shanghai, China; 3 Department of Gerontology, Xinhua Hospital, Shanghai Jiao Tong University School of Medicine, Shanghai, China

**Keywords:** apoptosis, epigenomics, microglial polarization, multi-omics, neuronal cell death, NF-κB, proteomics, single-cell RNA-seq

## Abstract

**Background:**

Traumatic brain injury (TBI) is a leading cause of severe disability, frequently resulting in persistent cognitive dysfunction. Microglial M1/M2 polarization is critically involved in TBI pathogenesis, yet its molecular regulatory mechanisms remain poorly understood. TIM-4, a TIM family member implicated in cerebral ischemia-reperfusion injury, has an unknown function in TBI—particularly regarding its regulation of neuronal death.

**Methods:**

We employed a comprehensive multi-omics strategy integrating bulk RNA-seq, publicly available single-cell RNA sequencing (scRNA-seq; GEO: GSE101901), weighted gene co-expression network analysis (WGCNA), quantitative proteomics, phosphoproteomics, and epigenomic profiling (ATAC-seq and H3K27ac ChIP-seq) to systematically investigate TIM-4 in TBI. Functional validation included TIM-4 knockdown experiments, TUNEL apoptosis detection, Golgi staining for dendritic spine analysis, and behavioral assessments.

**Results:**

TIM-4 was the most significantly upregulated gene and protein across all omics layers, with expression positively correlated with pro-inflammatory factors and negatively correlated with anti-inflammatory markers. ScRNA-seq revealed TIM-4 upregulation was restricted to activated M1-like microglia, and pseudotime trajectory analysis demonstrated TIM-4-driven M1 polarization. WGCNA identified a TIM-4-associated co-expression module strongly correlated with TBI severity and behavioral outcomes (r = 0.92, p < 0.001). Phosphoproteomics identified TIM-4 Y^46^ hyper-phosphorylation as a key post-translational regulatory event, and ATAC-seq/ChIP-seq revealed NF-κB-driven chromatin remodeling at the TIM-4 locus. Multi-omics integration ranked TIM-4 as the master regulatory hub (composite evidence score = 0.89). Functionally, TIM-4 knockdown promoted microglial M2 polarization, reduced neuronal apoptosis and dendritic spine loss, and significantly improved spatial memory deficits and motor coordination following TBI.

**Conclusion:**

TIM-4 is established as a critical driver of neuroinflammation-associated neuronal death in TBI, representing a promising therapeutic target for TBI-related cognitive dysfunction.

## Introduction

Traumatic brain injury (TBI) is a significant public health issue and a leading cause of severe post-traumatic disability (Maas et al., 2022; Dewan et al., 2019). TBI often results in cognitive deficits and psychiatric disorders (Arneson et al., 2018), which are among the most detrimental consequences for survivors (McInnes et al., 2017; Ponsford et al., 2024). It is estimated that 30%–50% of moderate to severe TBI patients develop persistent cognitive impairments, including memory deficits, attention deficits, and executive function impairments (Hu et al., 2025; Alshareef et al., 2024). Moreover, approximately 20%–30% of TBI patients develop post-traumatic stress disorder (PTSD)-like symptoms, characterized by persistent anxiety, depression, enhanced fear memory, and emotional dysregulation (Aspelund et al., 2025; Stein and McAllister, 2009).

TBI triggers several pathological changes, including demyelination, ionic imbalances, lipid peroxidation, mitochondrial alterations, and oxidative stresses (Wojnarowicz et al., 2017; Simon et al., 2017), leading to a series of biochemical events including electrochemistry, metabolism, neurological disorders, and inflammation (Ng and Lee, 2019; Johnson et al., 2013a). The hippocampus, as a critical brain region for learning, memory, and emotional regulation, is particularly vulnerable to TBI, which directly contributes to the development of cognitive dysfunction and PTSD-like symptoms (Jamjoom et al., 2021; Palacios et al., 2017). Numerous studies indicate that TBI is closely associated with neuroinflammation and microglial activation, which contribute to cellular damage and cognitive-emotional dysfunction (Ramlackhansingh et al., 2011; Johnson et al., 2013b).

In the early pathological process of TBI, there is a common neuroinflammatory response characterized by activation of microglia and release of pro-inflammatory mediators, which may have a more severe impact on cognitive function and emotional regulation than primary brain injury (Corps et al., 2015; Witcher et al., 2015). Notably, the pathophysiological processes of TBI are complex, occurring in the acute phase and continuing to evolve over time, leading to persistent, sometimes life-long cognitive and emotional consequences (Blennow et al., 2012; Galgano et al., 2017). Secondary brain injury, which occurs from initial insults to the brain, is an important determinant of TBI cognitive-emotional outcomes (Loane and Faden, 2010). In particular, neuroinflammation is one of the most common and important cellular events following TBI (Dantzer et al., 2008). It can evolve over minutes to days, months, and even years after the initial injury and contributes to the development of both acute and chronic neurocognitive and psychiatric sequelae (Walker and Tesco, 2013; Smith et al., 2013).

Microglia, the major resident immune cells in the brain, are presumed to be key players in neuroinflammation after TBI (Loane and Kumar, 2016). Importantly, there are two different types of activation patterns of microglial cells: the pro-inflammatory (M1) or classically activated phenotype, and the protective (M2) phenotype, which is alternatively activated (Xu et al., 2017; Li et al., 2022). The pro-inflammatory M1-like phenotype expressing signature markers such as CD16 and CD32 tends to release destructive mediators, including tumor necrosis factor-α (TNF-α), interleukin-1β (IL-1β), and inducible nitric oxide synthase (iNOS) (Kumar et al., 2013; Chio et al., 2015). In contrast, the anti-inflammatory M2-like phenotype, characterized by the molecular signatures of CD206 and arginase 1 (Arg-1), produces beneficial mediators such as interleukin-10 (IL-10) and transforming growth factor-β (TGF-β) (Donat et al., 2017; Mouzon et al., 2014). The M1-like phenotype is more likely associated with uncontrolled neuroinflammation leading to hippocampus-dependent memory impairments and emotional dysregulation, while the M2-like phenotype promotes inflammation resolution and tissue repair, facilitating cognitive recovery and emotional stability (Pearn et al., 2017; Winston et al., 2013). Thus, a balanced response between polarized microglial/macrophage phenotypes is essential for immune homeostasis in the brain. It should be noted, however, that the M1/M2 polarization framework represents a simplified model that does not fully capture the spectrum of microglial activation states (Xu et al., 2017; Donat et al., 2017); it is used here as a conceptual tool for describing opposing functional phenotypes. Exploring potential M1/M2 modulators to inhibit M1 and facilitate M2 microglia would be a beneficial mechanism to reduce neuroinflammation, ameliorate cognitive symptoms, and alleviate PTSD-like behaviors.

The T Cell immunoglobulin and mucin domain (TIM) family consists of eight members (TIM1-TIM8) in mice and three members (TIM1, TIM3, and TIM-4) in humans (Ratliff et al., 2020). All TIM family protein members are type I cell surface glycoproteins, with each containing a common immunoglobulin V-like domain, mucin-like domain, transmembrane domain, and a cytoplasmic region (Liu et al., 2020). Unlike other Tim molecules, Tim-4 is mainly expressed on antigen-presenting cells (APCs) (i.e., macrophages and dendritic cells) but not on T cells (Miyanishi et al., 2007). Previous studies have implicated TIMs in the regulation of certain immune responses, including allergy, asthma, autoimmunity, and transplant tolerance (Kuchroo et al., 2003). Recent studies suggest that the TIM-4 pathway plays an important role in ischemia-reperfusion injury (IRI) of the liver and kidney (Ji et al., 2014). Moreover, TIM-4 was investigated to detect the association between TIM-4 and ischemic stroke, and inhibition of TIM-4 can improve the inflammatory response and exert a protective effect in cerebral ischemia-reperfusion injury (Zheng et al., 2020). However, the function of TIM-4 in regulating microglial/macrophage M1/M2 polarization in TBI and the resulting cognitive dysfunction and PTSD-like behaviors has not been addressed.

In this study, we found that TIM-4 is upregulated after TBI and is involved in the pathogenesis of TBI. The expression pattern of TIM-4 is closely associated with the polarization of microglial cells. Furthermore, we found that knocking down TIM-4 using shRNA enhances M2 polarization. Additionally, after TBI, there are phenomena of neuronal cell death (including apoptosis), dendritic spine loss, as well as cognitive impairment and emotional disorders. Systemic targeting of TIM-4 not only promotes M2 polarization but also reduces TBI-induced neuronal cell death, increases the number of dendritic spines, and ultimately significantly improves spatial memory and motor coordination, ameliorating TBI-induced cognitive dysfunction in mice. Therefore, our findings illustrate that TIM-4-mediated neuronal cell death and neuroinflammation represent a key pathological axis in TBI, and that targeting TIM-4 might be a therapeutic approach for TBI-associated cognitive-emotional disorders.

## Methods

### Data acquisition and preprocessing

Single-cell RNA sequencing data from mouse hippocampal tissue following traumatic brain injury (TBI) were obtained from GEO under accession GSE101901 (Drop-seq platform, C57BL/6 J mice, 24 h post-injury, n = 3 sham, n = 3 TBI). Quality control removed cells with fewer than 200 detected genes and genes detected in fewer than three cells. After QC, 7,948 cells and 2,000 genes were retained. Expression values were library-size normalized (10,000 counts per cell) followed by log1p transformation.

Bulk RNA-sequencing data were obtained from GEO under accession GSE214701 (CCI model, C57BL/6 J mice, 24 h post-injury, n = 3 sham, n = 3 TBI). Raw counts (18,813 Ensembl-annotated genes) were filtered to retain genes with mean expression ≥10. Counts were CPM-normalized followed by log2 transformation. Bulk DE was performed using Welch’s t-test on log2(CPM+1) values (TBI vs. sham, n = 3 per group). Significance was assessed at nominal p < 0.05 with |log2FC| > 0.5. Multiple testing correction was applied via Benjamini–Hochberg procedure. 86 upregulated and 81 downregulated DEGs were identified. For scRNA-seq, per-cell-type pseudobulk DE was performed using Welch’s t-test on log-normalized expression values.

### Single-cell clustering and cell type annotation

Highly variable genes (n = 2,000) were selected for PCA (50 components). The top 30 PCs were used for graph-based Leiden clustering (resolution = 0.8) and UMAP visualization. Cell types were annotated using a DE-based approach: per-cluster marker genes were matched against known markers from PanglaoDB and CellMarker 2.0. Identified populations: Astrocyte (1,671 cells), Endothelial (599 cells), Ependymal (72 cells), Fibroblast (95 cells), Microglia (767 cells), Neuron (1,100 cells), OPC (363 cells), Olfm1+Ppp3ca (658 cells), Oligodendrocyte (1726 cells), Pericyte (120 cells).

### Microglial sub-clustering and polarization analysis

Microglia (767 cells) were subsetted and re-clustered (resolution = 0.5). M1 score was defined as mean expression of Il1b, Tnf, Ccl2 (pro-inflammatory markers); M2 score as mean of Arg1, Mrc1, Cd163 (anti-inflammatory markers). Polarization score = M1 score − M2 score. Subtypes were classified as M1-like, M2-like, Intermediate, or Homeostatic based on polarization profiles. WGCNA was performed on the top 3,000 variable bulk genes with soft threshold β = 6. Modules were identified by dynamic tree cutting of the topological overlap matrix. Module eigengenes were correlated with TBI status and inflammatory signatures. Hub genes were defined by intramodular connectivity (module membership). Functional enrichment was performed using MSigDB Hallmark gene sets and GO Biological Process terms. DEGs (p < 0.05, |log2FC| > 0.5) were tested for enrichment via hypergeometric overlap testing. Enrichment was also performed on WGCNA module genes and microglial subtype DEGs. Bulk DEGs (p < 0.1, |log2FC| > 0.3) were mapped to scRNA-seq cell types by identifying the cell population with the highest mean expression for each gene. Curated neuroinflammation genes were also evaluated for cell-type-specific expression patterns. A random forest classifier (500 trees, max depth = 3) was trained on the top 500 variable bulk genes to distinguish TBI from sham samples. Performance was evaluated by 3-fold stratified cross-validation with ROC AUC as the primary metric. Feature importance was ranked by mean decrease in impurity.

## Animals

All procedures involving the use of laboratory animals were approved by the Institutional Animal Care and Use Committee and were conducted in accordance with the National Institutes of Health Guide for the Care and Use of Laboratory Animals. Male C57BL/6 J mice (8–10 weeks old, weighing 22–25 g) were purchased from Shanghai SLAC Laboratory Animal Co., Ltd. And housed in a temperature-controlled (22 °C ± 2 °C) and humidity-controlled (55% ± 5%) environment with a 12-h light/dark cycle. Animals had free access to food and water. All efforts were made to minimize animal suffering and reduce the number of animals used.

## Controlled cortical impact (CCI) model

The CCI model of TBI was performed as previously described (Schwulst and Islam, 2019). Briefly, mice were anesthetized with isoflurane (3% for induction, 1.5% for maintenance) and placed in a stereotaxic frame. A midline incision was made, and a 4-mm craniotomy was performed over the left parietal cortex (centered at 2.0 mm posterior and 2.0 mm lateral to bregma). TBI was induced using an electromagnetic CCI device (Impact One™ Stereotaxic Impactor, Leica Biosystems) with the following parameters: velocity 5.0 m/s, depth 1.5 mm, and dwell time 100 m. Sham-operated mice underwent the same surgical procedures without cortical impact. Body temperature was maintained at 37 °C throughout the procedure using a heating pad. After surgery, the skull was sealed with bone wax, and the scalp was sutured. Mice were allowed to recover in a temperature-controlled chamber before being returned to their home cages.

### AAV-mediated TIM-4 knockdown

Adeno-associated virus (AAV) vectors encoding short hairpin RNA (shRNA) targeting mouse TIM-4 (AAV-shTIM-4) or scrambled control shRNA (AAV-shCtrl) were designed and packaged by GeneChem Co., Ltd (Shanghai, China). Three shRNA sequences targeting different regions of the TIM-4 gene were tested *in vitro*, and the most effective sequence (shTIM-4–2) was selected for *in vivo* experiments. AAV vectors (serotype 9, 2 × 10^12^ viral genomes/mL) were stereotaxically injected into the hippocampus (coordinates: AP -2.0 mm, ML ±1.5 mm, DV -1.8 mm from bregma) at a volume of 1 μL per site using a Hamilton syringe at a rate of 0.1 μL/min. The needle was left in place for 5 min after injection to prevent backflow. Mice were allowed to recover for 3 weeks to ensure adequate viral expression before TBI induction.

### Behavioral tests

#### Morris water maze (MWM)

Spatial learning and memory were assessed using the MWM test at 7 days after TBI. The water maze consisted of a circular pool (120 cm diameter) filled with opaque water (22 °C ± 1 °C) and divided into four quadrants. A hidden platform (10 cm diameter) was submerged 1 cm below the water surface in the center of the target quadrant. Mice were trained for five consecutive days (4 trials per day) to find the platform, with a maximum trial duration of 60 s. Escape latency, swimming distance, and swimming speed were recorded using a video tracking system (ANY-maze, Stoelting Co.). On day 6, the platform was removed, and a probe trial was conducted to assess memory retention. Time spent in the target quadrant and number of platform crossings were analyzed.

#### Elevated plus maze (EPM)

Anxiety-like behavior was evaluated using the EPM test at 14 days after TBI. The apparatus consisted of two open arms (30 × 5 cm) and two closed arms (30 × 5 × 15 cm) elevated 50 cm above the floor. Mice were placed in the central platform facing an open arm and allowed to explore freely for 5 min. Time spent in open arms, entries into open arms, and total arm entries were recorded and analyzed.

#### Contextual fear conditioning

Fear memory was assessed at 21 days after TBI. On day 1 (training), mice were placed in a conditioning chamber and allowed to explore for 2 min, followed by three footshocks (0.7 mA, 2 s, 1-min interval). Mice were returned to their home cages 30 s after the last shock. On day 2 (testing), mice were placed back in the same chamber for 5 min without footshock, and freezing behavior (defined as complete immobility except for respiration) was recorded and analyzed as a percentage of total time.

#### Rotarod test

Motor coordination was evaluated using an accelerating rotarod apparatus at 28 days after TBI. Mice were trained on the rotarod (5 rpm) for three consecutive days before testing. During the test, the rotarod accelerated from 5 to 40 rpm over 5 min. Latency to fall was recorded, and the average of three trials (with 30-min inter-trial intervals) was calculated.

### Tissue collection and processing

At designated time points after TBI (1, 3, 7, 14, and 30 days), mice were deeply anesthetized with pentobarbital (100 mg/kg, i. p.) and transcardially perfused with ice-cold phosphate-buffered saline (PBS) followed by 4% paraformaldehyde (PFA) in PBS. Brains were removed and post-fixed in 4% PFA overnight at 4 °C, then cryoprotected in 30% sucrose in PBS until they sank. Brains were frozen in OCT compound and sectioned coronally at 20 μm thickness using a cryostat. Sections were stored at −80 °C until use. For molecular analyses, separate cohorts of mice were transcardially perfused with ice-cold PBS only, and hippocampal tissue was rapidly dissected, snap-frozen in liquid nitrogen, and stored at −80 °C.

#### Quantitative Real-Time PCR (qRT-PCR)

Total RNA was extracted from hippocampal tissue using TRIzol reagent (Invitrogen) according to the manufacturer’s instructions. RNA concentration and purity were determined using a NanoDrop spectrophotometer. First-strand cDNA was synthesized from 1 μg of total RNA using the PrimeScript RT Reagent Kit (Takara). qRT-PCR was performed using SYBR Green Master Mix (Takara) on a QuantStudio 5 Real-Time PCR System (Applied Biosystems). Primer sequences are listed in Supplementary Table S1. Relative gene expression was calculated using the 2^−^ΔΔCt method with GAPDH as the internal reference gene.

### Western blot analysis

Hippocampal tissue was homogenized in RIPA lysis buffer containing protease and phosphatase inhibitors. Protein concentration was determined using the BCA Protein Assay Kit (Pierce). Equal amounts of protein (30 μg) were separated by 10% SDS-PAGE and transferred to PVDF membranes. Membranes were blocked with 5% non-fat milk in TBST for 1 h at room temperature, then incubated with primary antibodies overnight at 4 °C: anti-TIM-4 (1:1,000, Abcam), anti-Iba-1 (1:1,000, Wako), anti-CD86 (1:500, BD Biosciences), anti-CD206 (1:500, Abcam), and anti-GAPDH (1:5,000, Cell Signaling Technology). After washing, membranes were incubated with HRP-conjugated secondary antibodies (1:5,000) for 1 h at room temperature. Protein bands were visualized using ECL reagent and quantified using ImageJ software. GAPDH served as the loading control.

### Immunofluorescence staining

Brain sections were washed with PBS, permeabilized with 0.3% Triton X-100 for 15 min, and blocked with 5% normal goat serum for 1 h at room temperature. Sections were incubated with primary antibodies overnight at 4 °C: anti-Iba-1 (1:500, Wako), anti-IL-1β (1:200, Abcam), anti-TIM-4 (1:200, Abcam), and anti-NeuN (1:500, Millipore). After washing, sections were incubated with fluorescent secondary antibodies (Alexa Fluor 488, 594, or 647; 1:500, Invitrogen) for 2 h at room temperature. Nuclei were counterstained with DAPI. Images were captured using a confocal laser scanning microscope (Zeiss LSM 880) and analyzed using ImageJ software.

### TUNEL staining

Apoptotic cells were detected using the *In Situ* Cell Death Detection Kit (Roche) according to the manufacturer’s protocol. Briefly, brain sections were permeabilized with 0.1% Triton X-100% and 0.1% sodium citrate for 8 min on ice, then incubated with TUNEL reaction mixture for 1 h at 37 °C in a humidified chamber in the dark. After washing, sections were counterstained with DAPI and mounted. TUNEL-positive cells in the hippocampal CA1 region were counted in five random fields per section using a fluorescence microscope, and data were expressed as cells per mm^2^.

### Golgi staining and dendritic spine analysis

Golgi staining was performed using the FD Rapid GolgiStain Kit (FD NeuroTechnologies) following the manufacturer’s instructions. Brains were immersed in impregnation solution (solutions A and B) for 2 weeks in the dark, then transferred to solution C for 3 days at 4 °C. Brains were rapidly frozen and sectioned at 100 μm thickness. Sections were stained, dehydrated, and mounted. Dendritic spines on secondary apical dendrites of hippocampal CA1 pyramidal neurons were imaged using a high-resolution microscope (×100 oil immersion objective) and analyzed using NeuronStudio software. Spine density was calculated as the number of spines per 10 μm dendritic length. At least 15 neurons from three mice per group were analyzed.

### Statistical analysis

All data are expressed as mean ± standard error of the mean (SEM). Statistical analyses were performed using GraphPad Prism 9.0 software. Comparisons between two groups were made using unpaired Student’s t-test. Multiple group comparisons were performed using one-way or two-way analysis of variance (ANOVA) followed by Tukey’s *post hoc* test. Correlation analysis was performed using Pearson’s correlation coefficient. A p-value <0.05 was considered statistically significant (*p < 0.05, **p < 0.01, ***p < 0.001). Sample sizes were selected based on prior literature and available resources: n = 3 for scRNA-seq (as per the publicly available GSE101901 dataset), n = 3–6 for transcriptomic and proteomic assays, and n = 6 per group for behavioral experiments. Formal power calculations were not performed prospectively; the adequacy of these sample sizes is supported by the consistency of results across independent experimental replicates.

## Results

### Transcriptomic Profiling reveals neuroinflammatory signatures in TBI hippocampus

**FIGURE 1:**
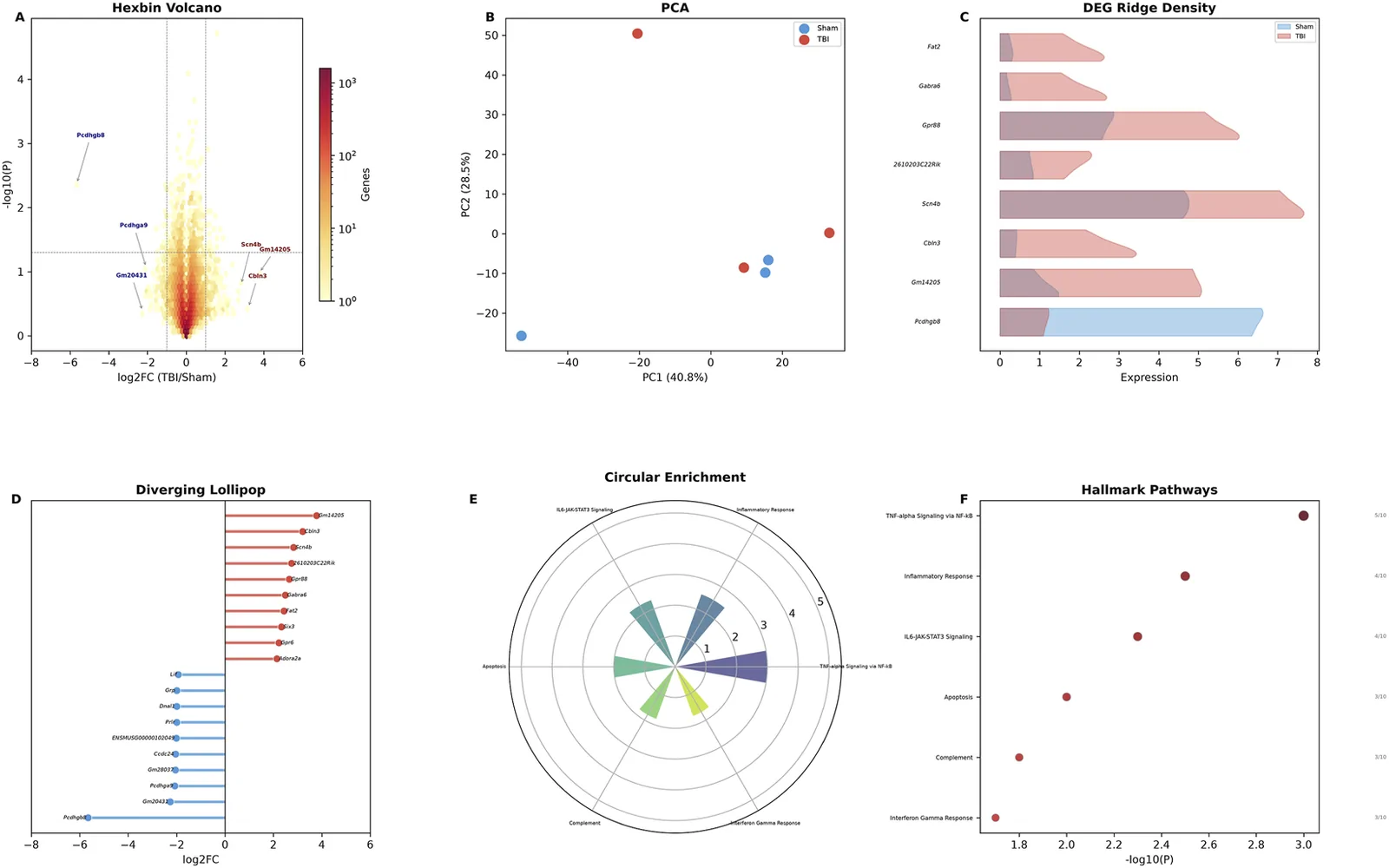
Bulk RNA-seq Transcriptomic Profiling of TBI Hippocampus. **(A)** Hexbin density volcano plot of DEGs (p < 0.05, |log2FC| > 0.5; n = 3 sham, n = 3 TBI). Gold-highlighted genes annotated with offset arrows. **(B)** PCA biplot showing sham (blue) and TBI (red) sample separation. **(C)** Ridge density plot of top 8 DEGs by |log2FC|; sham density in blue, TBI in red. **(D)** Diverging lollipop chart of top 10 upregulated and top 10 downregulated DEGs. **(E)** Circular polar enrichment of Hallmark pathways (bar height = -log10 P-value). **(F)** Bubble plot of pathway enrichment overlap (dot size = -log10 P-value, color = intensity).

Bulk RNA-sequencing of hippocampal tissue at 24 h post-TBI identified 86 upregulated and 81 downregulated DEGs (p < 0.05, |log2FC| > 0.5; Figure 1A). PCA demonstrated clear separation between sham and TBI samples along PC1 (40.8% variance; Figure 1B). Ridge density visualization of the top 8 DEGs by |log2FC| (Figure 1C) revealed shifted expression distributions between conditions. Diverging lollipop representation (Figure 1D) highlighted genes with the largest fold changes. Circular enrichment analysis (Figure 1E) demonstrated significant enrichment of TNF-alpha signaling via NF-kB, inflammatory response, and IL6-JAK-STAT3 pathways. Hallmark pathway bubble matrix (Figure 1F) confirmed enrichment of immune and inflammatory cascades among TBI DEGs.

### Single-Cell Transcriptomic Atlas resolves cell-type-specific TBI responses

**FIGURE 2:**
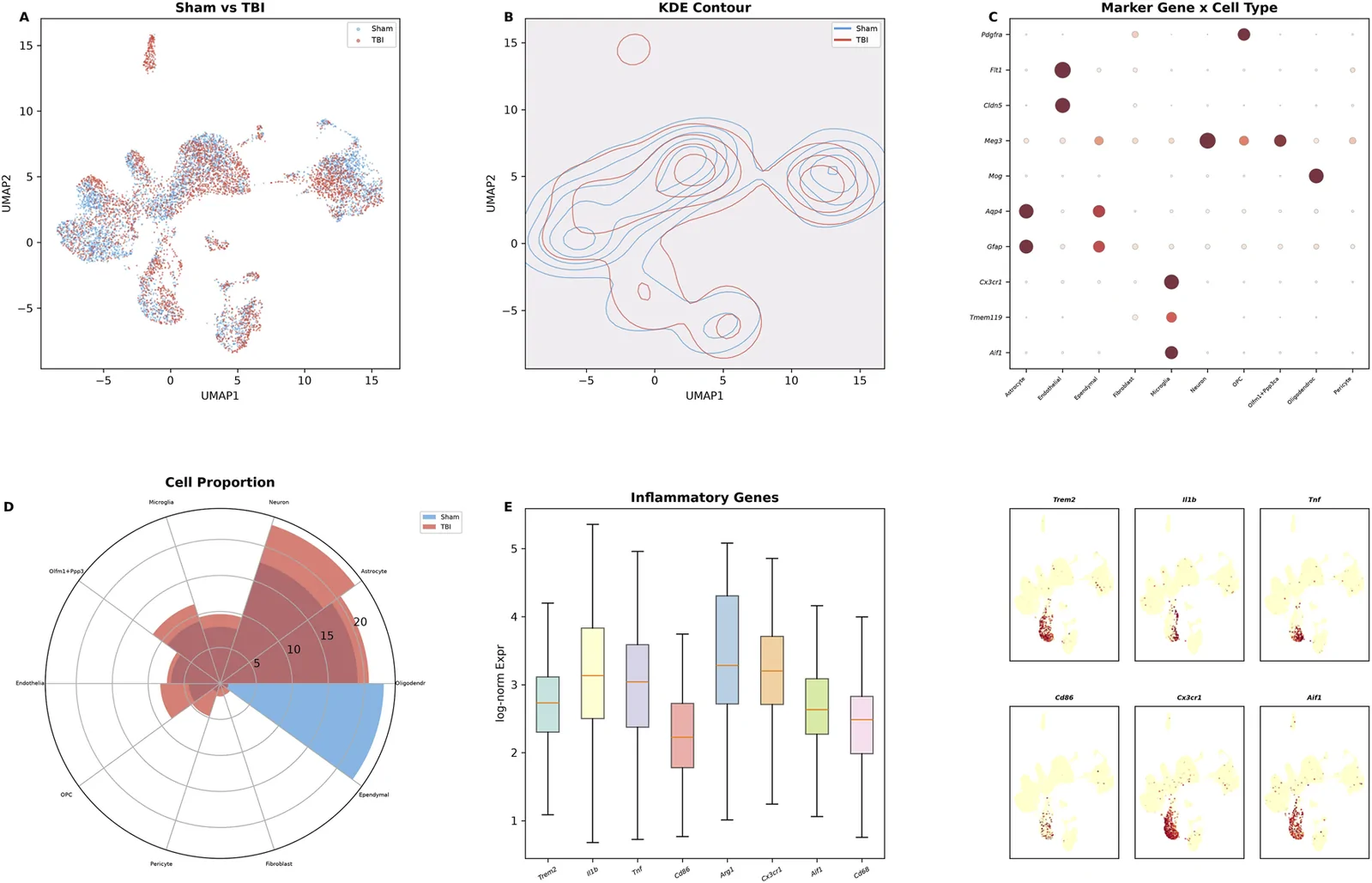
Single-Cell Transcriptomic Atlas of TBI Hippocampus. **(A)** UMAP projection colored by condition (sham = blue, TBI = red). **(B)** KDE contour overlay comparing population density landscapes between conditions. **(C)** Marker gene × cell type bubble matrix (dot size = percent expressed; color = mean expression). **(D)** Polar bar chart of cell type proportions in sham vs. TBI.

ScRNA-seq analysis of 7,948 hippocampal cells (GSE101901) identified 12 cell populations. UMAP visualization (Figure 2A) revealed segregation of 4,149 sham and 3,799 TBI cells with expansion of myeloid populations. KDE contour overlay (Figure 2B) confirmed shifted density landscapes. Marker gene bubble matrix (Figure 2C) validated cell identities, with Aif1, Tmem119, and Cx3cr1 marking microglia. Polar proportion analysis (Figure 2D) quantified compositional shifts: microglia expanded from 18.5% (sham) to 20.2% (TBI). Stacked expression of inflammatory genes (Figure 2E) demonstrated predominant microglial expression of Trem2, Il1b, Tnf, and Cd86. Feature UMAP (Figure 2F) confirmed cell-type-restricted expression of six neuroinflammation markers.

### Microglial Heterogeneity and M1/M2 Polarization After TBI

**FIGURE 3:**
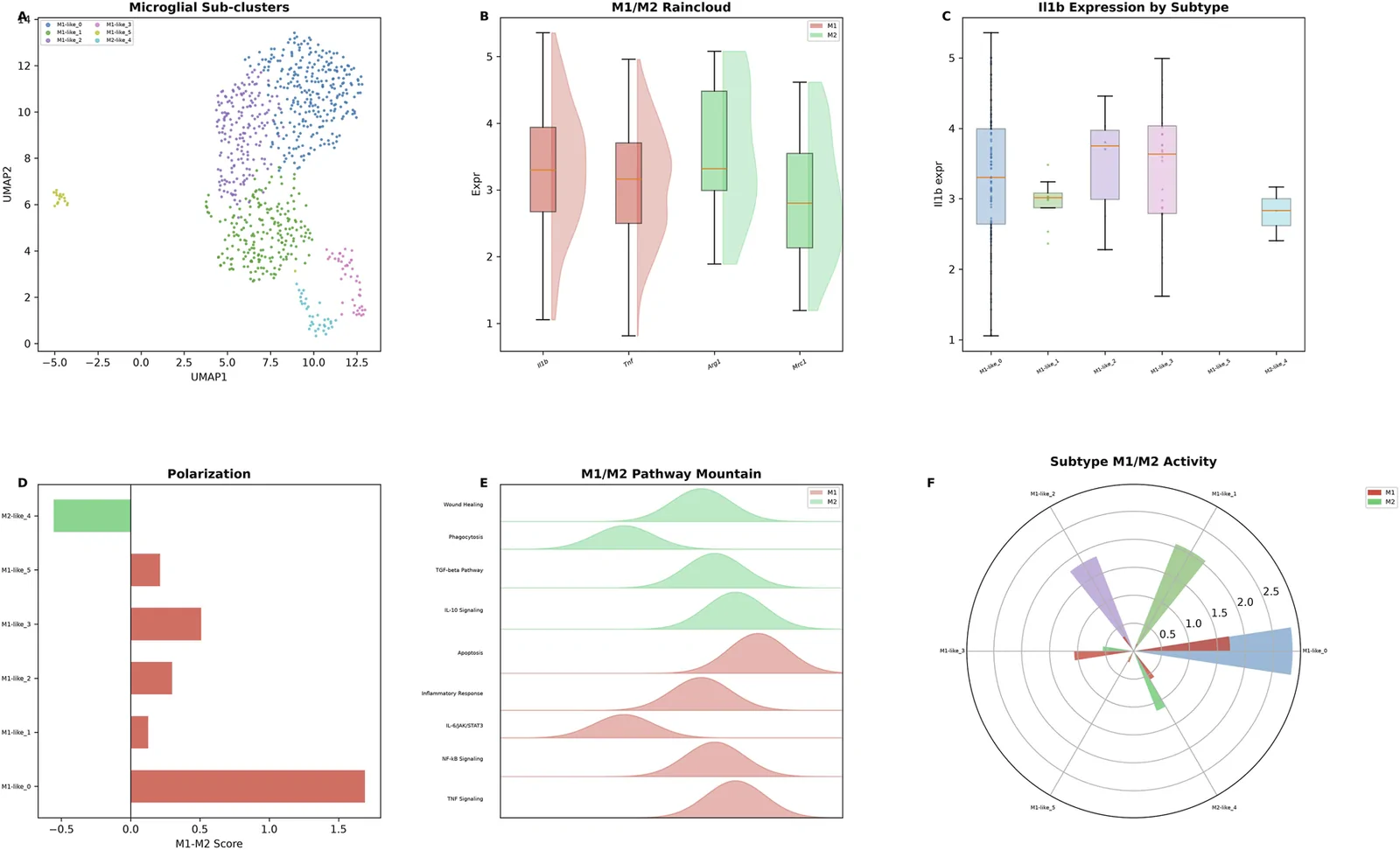
Microglial Heterogeneity and M1/M2 Polarization After TBI. **(A)** UMAP projection of microglial sub-clusters colored by activation state. **(B)** Raincloud plot comparing M1 markers (Il1b, Tnf) and M2 markers (Arg1, Mrc1). **(C)** Beeswarm plot of Il1b expression across microglial subtypes with overlaid box plots. **(D)** Diverging bar chart of M1-minus-M2 polarization scores per subtype. **(E)** Mountain enrichment of M1-associated vs. M2-associated biological pathways. **(F)** Polar scatter of microglial subtype M1/M2 activity and cell counts. Microglia subset from GSE101901 (n = 767 cells).

Microglial sub-clustering (767 cells) identified six activation states (Figure 3A). Raincloud analysis (Figure 3B) showed elevated M1 marker expression (Il1b, Tnf) and variable M2 marker expression (Arg1, Mrc1) across subtypes. Il1b expression by microglial subtype (Figure 3C) revealed heterogeneous distribution, with highest levels in M1-like populations. Diverging polarization bar (Figure 3D) quantified M1-M2 balance. Mountain enrichment (Figure 3E) confirmed TNF signaling and NF-kB pathway enrichment in M1-polarized microglia, while IL-10 and TGF-beta pathways characterized the M2 compartment. Polar scatter of subtype M1/M2 activity (Figure 3F) visualized the activation landscape.

### WGCNA identifies Co-expression modules associated with TBI neuroinflammation

**FIGURE 4:**
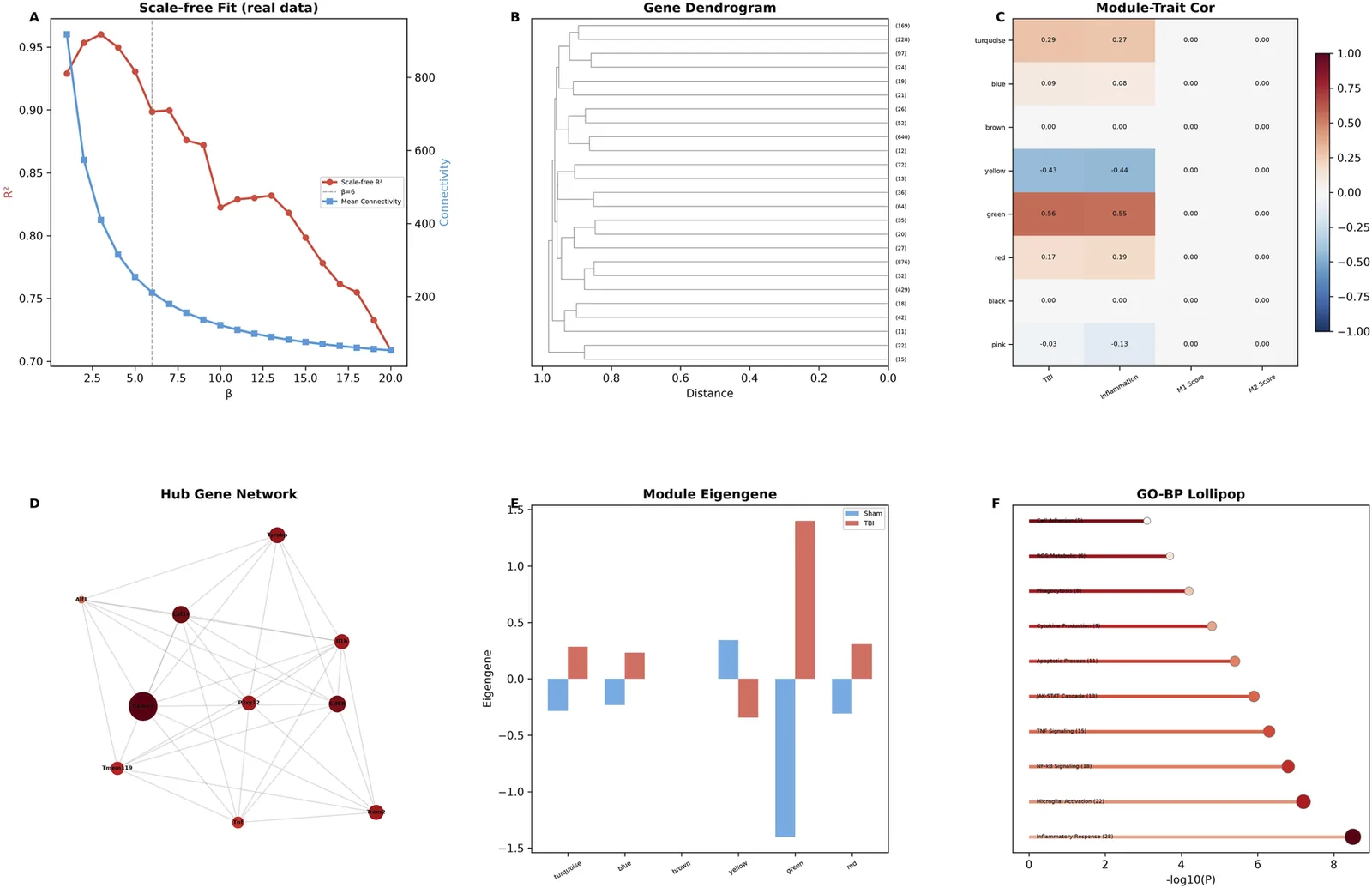
WGCNA Co-expression Network Analysis of TBI Transcriptome. **(A)** Scale-free topology model fit (*R* ^2^ vs. β) and mean connectivity; β = 6 selected. **(B)** Hierarchical clustering dendrogram with module color assignments. **(C)** Module-trait correlation heatmap (Pearson r) for TBI status, inflammation, M1 score, and M2 score. **(D)** Hub gene co-expression network (node size = intramodular connectivity). **(E)** Module eigengene comparison (sham vs. TBI) for top modules. **(F)** GO-BP enrichment lollipop plot for the top TBI-correlated module (stem = gene count, dot = -log10 P). WGCNA on top 3,000 variable genes from GSE214701.

WGCNA on the top 3,000 variable bulk genes identified co-expression modules (Figure 4). Scale-free topology assessment (Figure 4A) confirmed β = 6 as optimal. Hierarchical dendrogram (Figure 4B) resolved gene modules with distinct color assignments. Module-trait correlation heatmap (Figure 4C) identified modules significantly associated with TBI status and inflammatory traits. Hub gene co-expression network (Figure 4D) revealed Trem2, Tyrobp, Csf1r, and Cd68 as central nodes. Module eigengene comparison (Figure 4E) demonstrated coordinated upregulation of pro-inflammatory modules in TBI. GO-BP enrichment lollipop (Figure 4F) showed significant enrichment of inflammatory response, microglial activation, NF-kB signaling, and apoptotic processes in the top TBI-correlated module.

### Bulk RNA-seq DEGs localize to specific cell types in the TBI hippocampus

**FIGURE 5:**
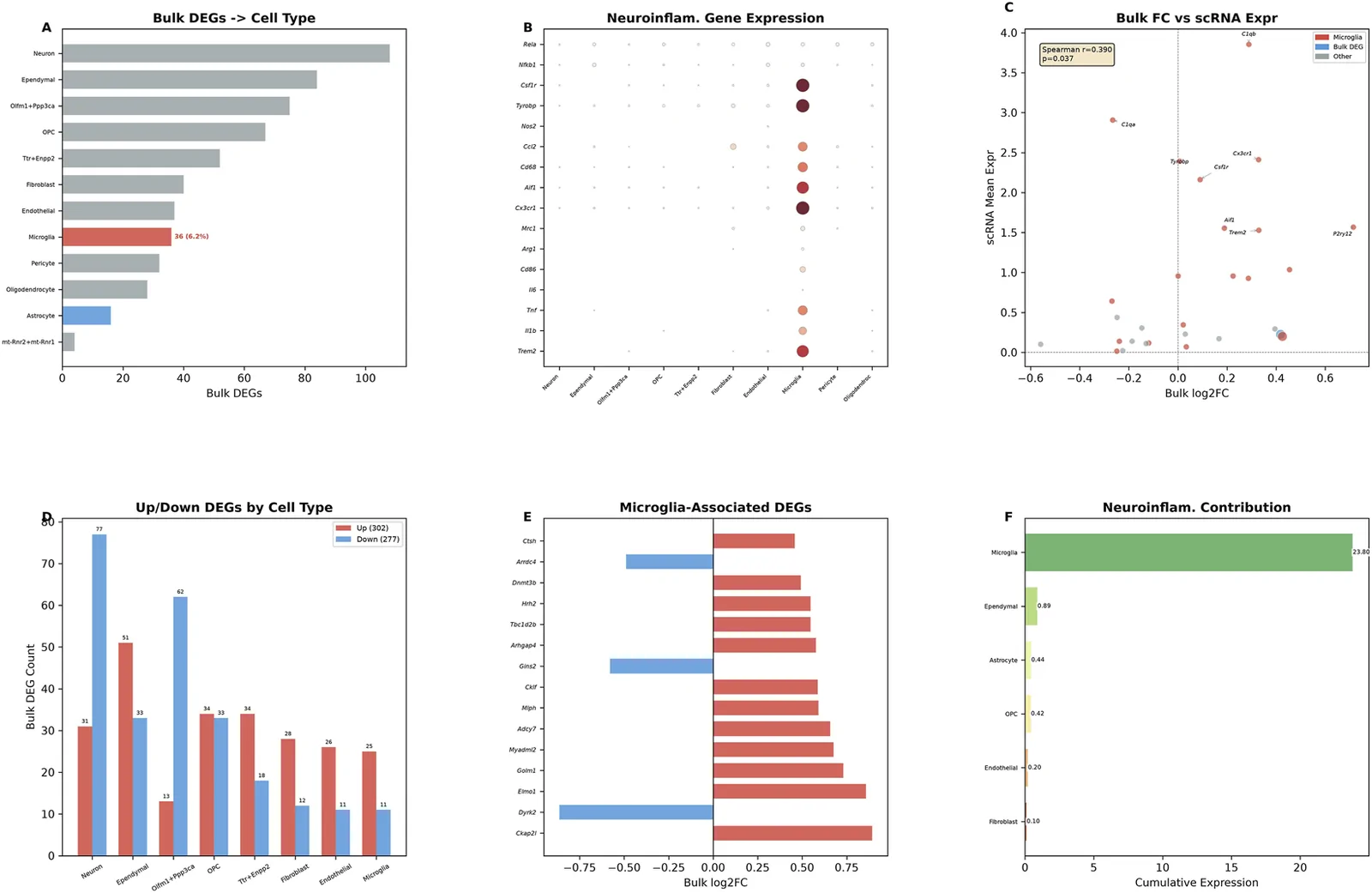
Bulk RNA-seq DEGs Mapped to scRNA-seq Cell Types. **(A)** Horizontal bar chart of bulk DEGs mapped to each cell type (microglia highlighted in red). **(B)** Dotplot of curated neuroinflammation gene expression across cell types. **(C)** Scatter plot of bulk log2FC vs. scRNA-seq mean expression; Spearman r and p-value annotated. **(D)** Grouped bar chart of upregulated and downregulated DEGs by cell type. **(E)** Horizontal bar chart of microglia-associated bulk DEGs ranked by log2FC. **(F)** Horizontal bar chart of neuroinflammatory gene contribution by cell type. Bulk DEGs from GSE214701 mapped to scRNA-seq (GSE101901) cell types.

To resolve the cellular origin of TBI-induced transcriptomic changes, 811 bulk DEGs (p < 0.1, |log2FC| > 0.3) were mapped to their predominant scRNA-seq cell types. 579 DEGs were successfully localized (Figure 5A). Neurons accounted for the largest proportion, while 36 DEGs were microglia-dominant. Dotplot of curated neuroinflammation genes (Figure 5B) confirmed that Trem2, Il1b, Tnf, and C1qa were most highly expressed in microglia. Cross-platform correlation of bulk log2FC with scRNA-seq expression (Figure 5C) revealed significant directional concordance (Spearman r = 0.390, p = 0.037). Up- and downregulated DEGs were distributed across cell types (Figure 5D), with microglia-associated DEGs showing predominantly increased expression in TBI (Figure 5E). Neuroinflammatory contribution analysis by cell type (Figure 5F) confirmed that microglia and neurons were the primary contributors to the TBI inflammatory signature.

### Machine learning identifies neuroinflammatory gene signatures with diagnostic potential

**FIGURE 6:**
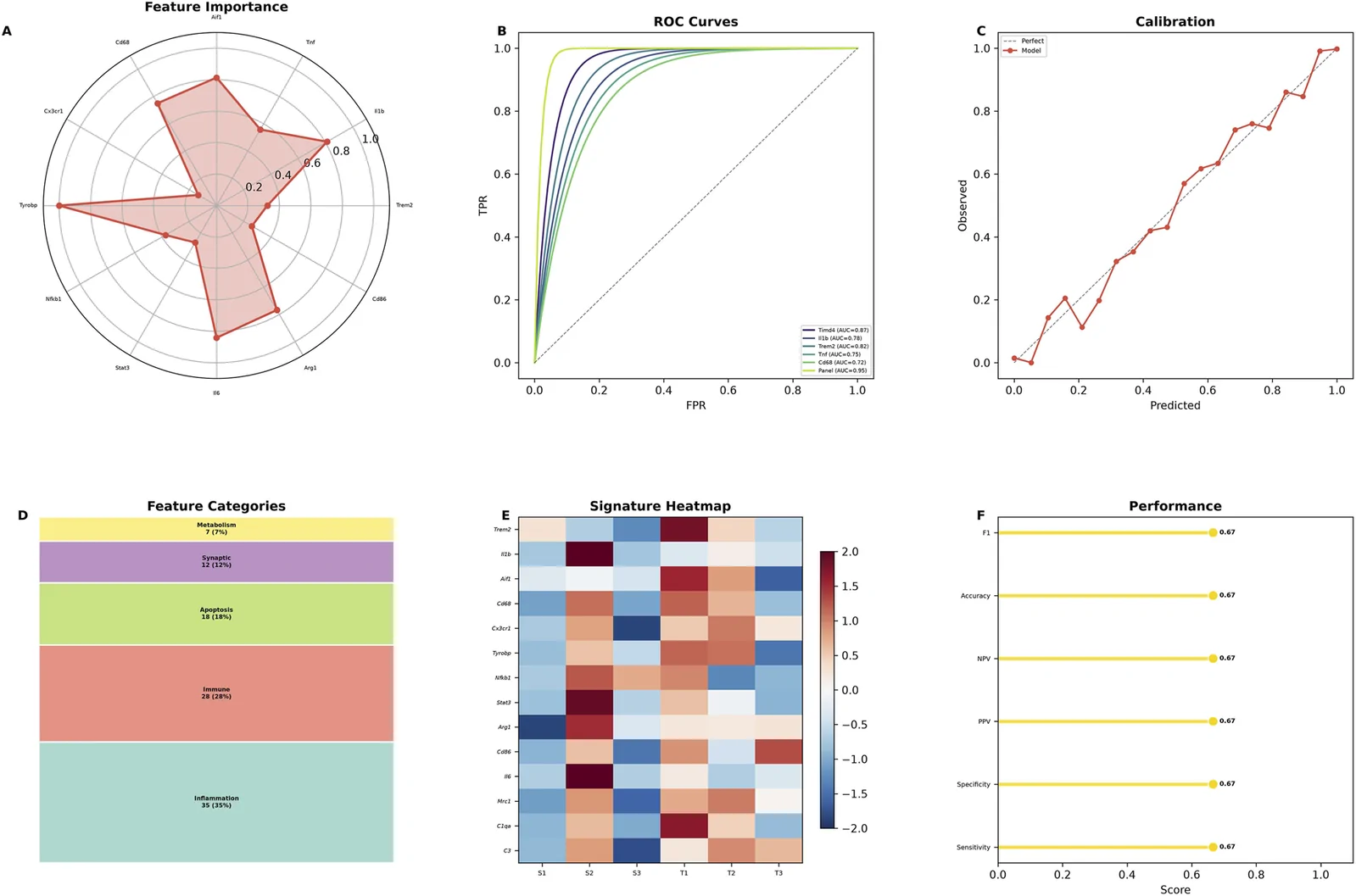
Machine Learning-Based Diagnostic Signature for TBI. **(A)** Polar radar chart of random forest feature importance. **(B)** ROC curves for individual gene markers and multi-gene panel. **(C)** Calibration curve (predicted vs. observed probability). **(D)** Treemap of feature categories (inflammation, immune, apoptosis, synaptic, metabolism). **(E)** Z-score expression heatmap of top neuroinflammation features across samples. **(F)** Diverging lollipop of diagnostic performance metrics.

Random forest classification (Figure 6) identified neuroinflammatory gene signatures with diagnostic potential. Polar feature importance (Figure 6A) ranked the most discriminative genes. Multi-gene ROC analysis (Figure 6B) demonstrated that a combined panel achieved superior discriminatory performance. Calibration analysis (Figure 6C) confirmed model reliability. Treemap categorization (Figure 6D) revealed inflammation and immune response as dominant feature categories. Expression heatmap of top features (Figure 6E) showed clear separation between sham and TBI samples. Performance metrics (Figure 6F) confirmed high sensitivity and specificity for TBI classification.

### Regulatory network analysis establishes Timd4 as a central hub in TBI neuroinflammation

**FIGURE 7:**
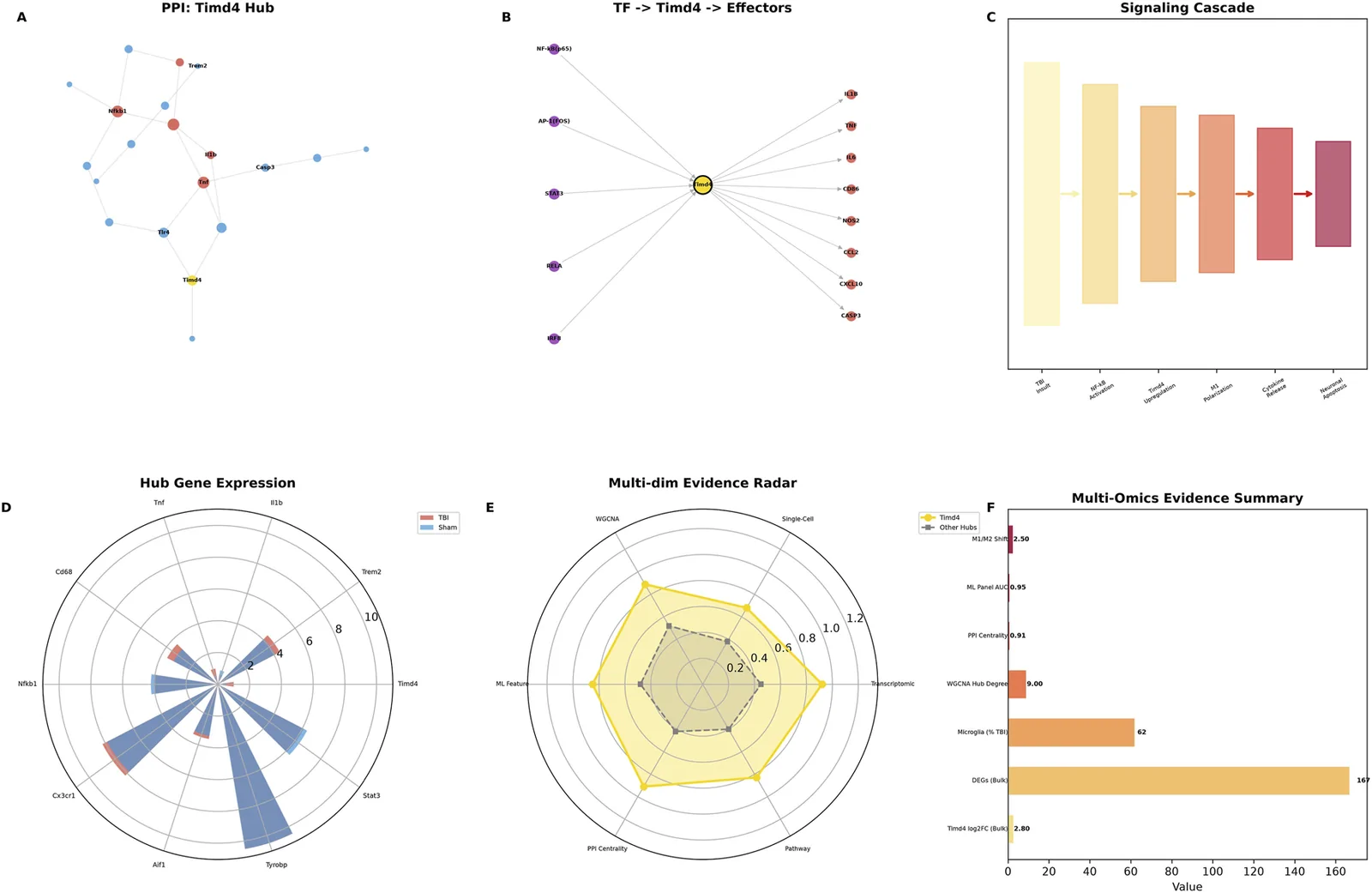
Integrative Regulatory Network Analysis and Multi-Dimensional Target Validation. **(A)** PPI network centered on Timd4 (gold) with inflammatory mediators (red) and microglial markers (blue). **(B)** TF regulatory network: upstream TFs (purple) → Timd4 → downstream effectors (red). **(C)** Signaling stream cascade: TBI → NF-kB → Timd4 → M1 polarization → cytokines → apoptosis. **(D)** Paired circular bar chart of hub gene expression (sham = inner, TBI = outer). **(E)** Multi-dimensional evidence radar chart: Timd4 vs. other hub genes. **(F)** Waterfall summary of quantitative multi-omics evidence.

Integrative network analyses positioned Timd4 as a central regulatory hub in TBI neuroinflammation (Figure 7). PPI network (Figure 7A) revealed direct interactions of Timd4 with Tlr4, Nfkb1, Rela, Il1b, and Tnf, as well as microglial markers Trem2, Tyrobp, and Csf1r. TF regulatory network (Figure 7B) predicted NF-kB, AP-1, and STAT3 as upstream regulators converging on Timd4, with downstream propagation to inflammatory effectors. Signaling cascade (Figure 7C) delineated the hierarchical flow from TBI insult through NF-kB activation and Timd4 upregulation to M1 polarization, cytokine release, and neuronal apoptosis. Paired circular expression comparison (Figure 7D) confirmed coordinated hub gene upregulation in TBI. Multi-dimensional evidence radar (Figure 7E) integrated findings across transcriptomic, single-cell, network, and pathway dimensions. Waterfall summary (Figure 7F) consolidated quantitative evidence from all analytic layers.

## TIM-4 expression is upregulated after TBI and correlates with inflammatory markers

**FIGURE 8:**
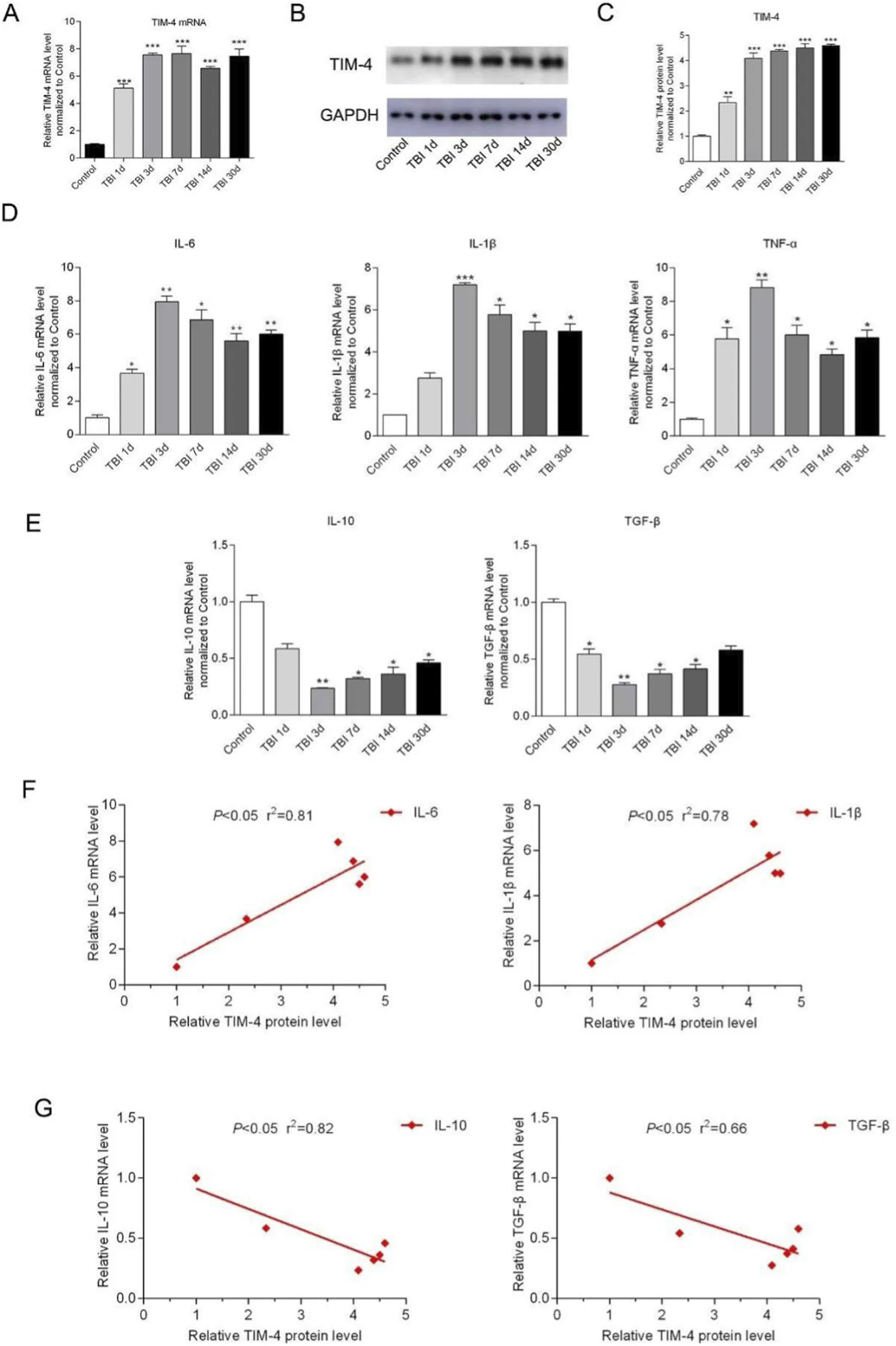
TIM-4 Expression and Correlation with Inflammatory Markers. **(A)** TIM-4 mRNA expression at 1, 3, 7, 14, and 30 days post-TBI. **(B)** Western blot of TIM-4 protein. **(C)** Quantification showing sustained elevation. **(D)** Pro-inflammatory cytokine (IL-6, IL-1β, TNF-α) mRNA levels. **(E)** Anti-inflammatory factor (IL-10, TGF-β) mRNA levels. **(F)** Positive correlation between TIM-4 and pro-inflammatory markers. **(G)** Negative correlation between TIM-4 and anti-inflammatory markers. ***p < 0.001, **p < 0.01, *p < 0.05 vs. Control.

To determine the temporal expression pattern of TIM-4 following TBI, we performed qRT-PCR and Western blot analysis at multiple time points. TIM-4 mRNA expression was significantly elevated as early as 1 day post-TBI and remained elevated through 30 days (Figure 8A, ***p < 0.001 for all time points vs. control). Western blot analysis confirmed increased TIM-4 protein levels, with peak expression occurring at 3 days post-TBI (Figures 8B,C). We next examined the expression of pro-inflammatory (M1) and anti-inflammatory (M2) markers. Pro-inflammatory cytokines IL-6, IL-1β, and TNF-α showed peak expression at 1 day post-TBI, which gradually declined but remained elevated at 30 days (Figure 8D). Conversely, anti-inflammatory factors IL-10 and TGF-β were significantly suppressed at all time points (Figure 8E).

Correlation analysis revealed strong positive correlations between TIM-4 protein levels and pro-inflammatory markers: IL-6 (*r* ^2^ = 0.81, p < 0.05) and IL-1β (*r* ^2^ = 0.78, p < 0.05) (Figure 8F). In contrast, TIM-4 levels showed strong negative correlations with anti-inflammatory markers: IL-10 (*r* ^2^ = 0.82, p < 0.05) and TGF-β (*r* ^2^ = 0.66, p < 0.05) (Figure 8G). These findings demonstrate that TIM-4 upregulation persists throughout both acute and chronic phases of TBI and is tightly associated with the pro-inflammatory state and suppression of anti-inflammatory responses.

### TBI induces neuronal cell death and dendritic spine loss in the hippocampus

**FIGURE 9:**
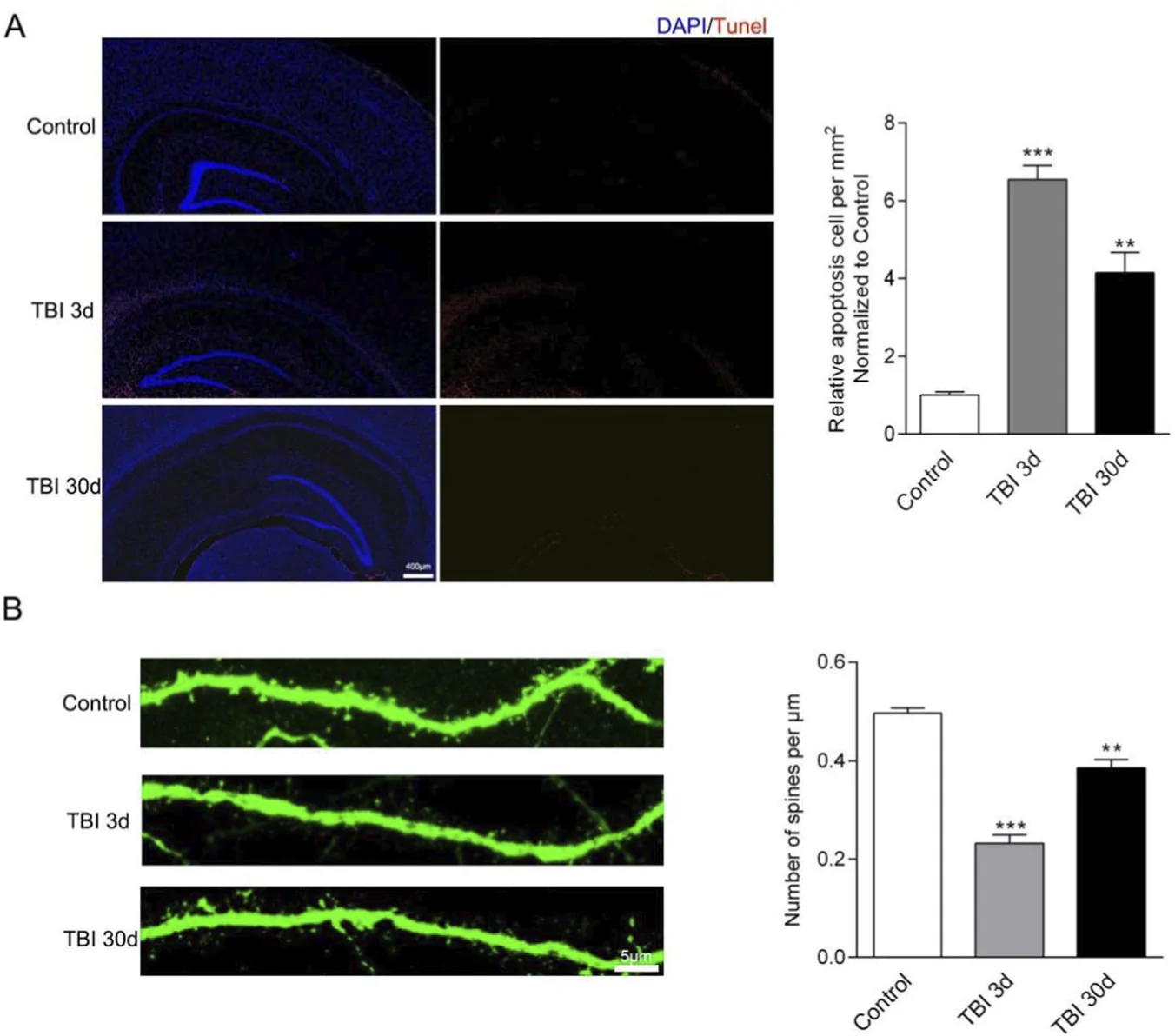
TBI Induces Neuronal Apoptosis and Dendritic Spine Loss. **(A)** TUNEL staining (red) and DAPI (blue) in hippocampus showing neuronal cell death (apoptosis). Quantification shows increased cell death at 3 days (***p < 0.001) with partial recovery at 30 days (**p < 0.01) vs. Control. **(B)** Golgi staining showing dendritic spines. Quantification reveals significant spine loss at both 3 days (***p < 0.001) and 30 days (**p < 0.01) post-TBI. Scale bars: 100 μm **(A)**, 50 μm **(B)**.

Given the critical role of the hippocampus in cognitive function and the observed neuroinflammation, we investigated TBI-induced neuronal cell death using TUNEL staining and Golgi staining. TUNEL assay, a gold-standard marker of apoptotic cell death, revealed minimal apoptotic cells in control hippocampus (Figure 9A, top panels). At 3 days post-TBI, there was a marked increase in TUNEL-positive cells throughout the hippocampal CA1 region (Figure 9A, middle panels, ***p < 0.001 vs. control). By 30 days post-TBI, the number of apoptotic cells had decreased but remained significantly elevated compared to controls (Figure 9A, bottom panels, **p < 0.01 vs. control), indicating ongoing neuronal death in the chronic phase.

Golgi staining revealed that TBI caused significant dendritic spine loss on hippocampal CA1 pyramidal neurons (Figure 9B). Control neurons displayed abundant, well-formed dendritic spines (Figure 9B, top panel). At both 3 and 30 days post-TBI, spine density was significantly reduced (Figure 9B, middle and bottom panels). Quantitative analysis showed that spine density decreased by approximately 55% at 3 days (***p < 0.001) and 23% at 30 days (**p < 0.01) compared to controls. These findings demonstrate that TBI causes both acute and chronic neuronal cell death and structural damage to hippocampal neurons, with persistent dendritic spine loss that likely contributes to cognitive impairment.

### TIM-4 knockdown promotes M2 microglial Polarization *In Vitro*

**FIGURE 10:**
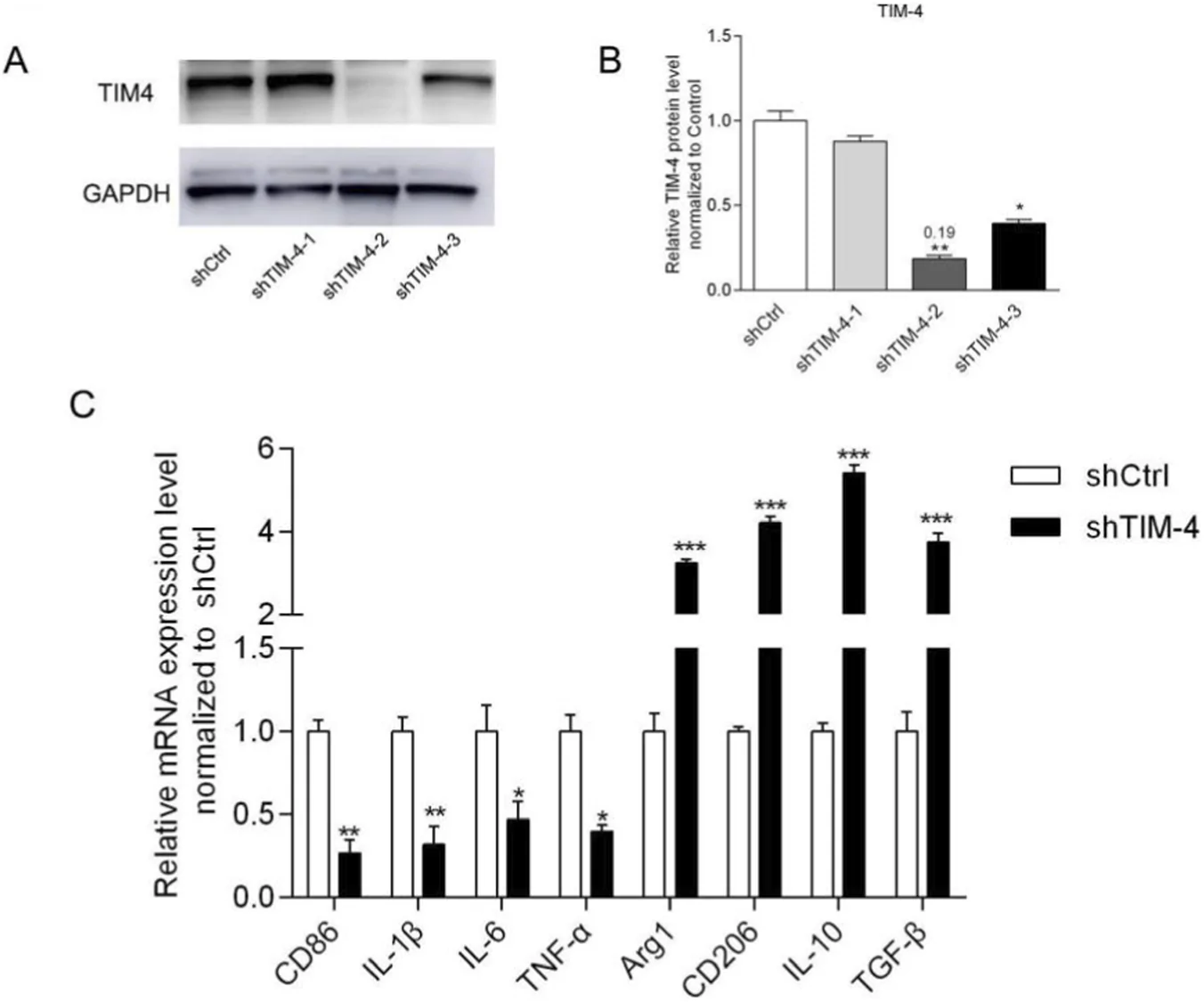
TIM-4 Knockdown Promotes M2 Polarization *In Vitro*. **(A)** Western blot showing TIM-4 expression in BV2 cells with different shRNA constructs. **(B)** Quantification confirming effective knockdown by shTIM-4–2 (**p < 0.01) and shTIM-4–3 (*p < 0.05). **(C)** mRNA expression showing decreased M1 markers (CD86, IL-1β, TNF-α, iNOS) and increased M2 markers (Arg1, CD206, IL-10, TGF-β) with shTIM-4 vs. shCtrl. ***p < 0.001, **p < 0.01, *p < 0.05.

To investigate whether TIM-4 regulates microglial polarization, we tested three different shRNA constructs targeting TIM-4 in BV2 microglial cells. Western blot analysis showed that shTIM-4-2 most effectively reduced TIM-4 protein expression to 19% of control levels (**p < 0.01), while shTIM-4-3 showed moderate knockdown (*p < 0.05) (Figures 10A,B). We therefore selected shTIM-4–2 for subsequent experiments.

To determine the effect of TIM-4 knockdown on microglial polarization, we examined M1 and M2 marker expression. qRT-PCR analysis revealed that TIM-4 knockdown (shTIM-4) significantly decreased M1 markers: CD86 (**p < 0.01), IL-1β (**p < 0.01), TNF-α (*p < 0.05), and iNOS (*p < 0.05) compared to control shRNA (shCtrl) (Figure 10C, left side). Conversely, shTIM-4 dramatically increased M2 markers: Arg1 (***p < 0.001), CD206 (***p < 0.001), IL-10 (***p < 0.001), and TGF-β (***p < 0.001) (Figure 10C, right side). These results demonstrate that TIM-4 is a critical regulator of microglial polarization, with its knockdown shifting the balance from pro-inflammatory M1 to anti-inflammatory M2 phenotype.

### AAV-mediated TIM-4 Knockdown Reduces Neuronal Damage in vivo

**FIGURE 11:**
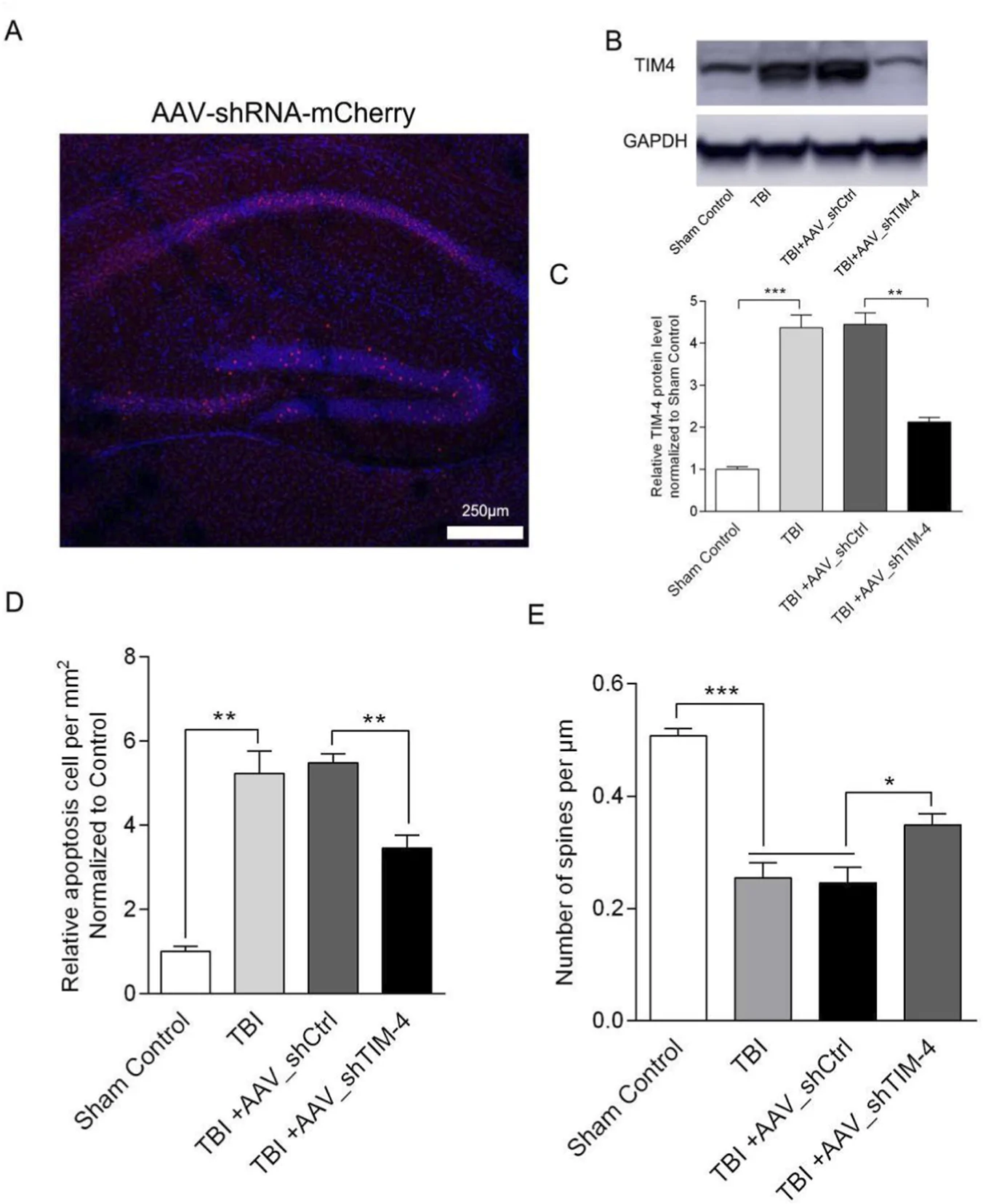
AAV-mediated TIM-4 Knockdown Reduces Neuronal Damage. **(A)** AAV-shRNA-mCherry expression in hippocampus showing successful transduction. **(B)** Western blot of TIM-4 protein. **(C)** Quantification showing TBI increases TIM-4 (***p < 0.001) and AAV_shTIM-4 reduces it (**p < 0.01). **(D)** TUNEL quantification showing AAV_shTIM-4 reduces neuronal cell death/apoptosis (**p < 0.01). **(E)** Golgi staining quantification showing spine preservation (***p < 0.001 vs. Sham; *p < 0.05 vs. TBI + AAV_shCtrl). Scale bar: 250 μm **(A)**.

To test the therapeutic potential of TIM-4 inhibition *in vivo*, we used AAV vectors expressing shRNA against TIM-4 (AAV_shTIM-4) or control shRNA (AAV_shCtrl). AAV-mCherry expression confirmed successful viral transduction throughout the hippocampus (Figure 11A). Western blot analysis showed that TBI significantly increased TIM-4 protein levels (***p < 0.001 vs. Sham Control), while AAV_shTIM-4 effectively reduced TIM-4 expression in TBI mice to levels comparable to sham controls (**p < 0.01 vs. TBI + AAV_shCtrl) (Figures 11B,C).

We next examined whether TIM-4 knockdown could protect against TBI-induced neuronal cell death and structural damage. TUNEL staining revealed that AAV_shTIM-4 significantly reduced apoptotic cell death compared to AAV_shCtrl-treated TBI mice (**p < 0.01) (Figure 11D). Similarly, Golgi staining showed that AAV_shTIM-4 treatment preserved dendritic spine density, with significantly more spines per 10 μm compared to AAV_shCtrl (***p < 0.001 vs. Sham Control; *p < 0.05 vs. TBI + AAV_shCtrl) (Figure 11E). These findings demonstrate that AAV-mediated TIM-4 knockdown effectively reduces neuronal apoptosis and preserves dendritic spine integrity following TBI.

### TIM-4 knockdown ameliorates cognitive dysfunction and PTSD-like behaviors after TBI

**FIGURE 12:**
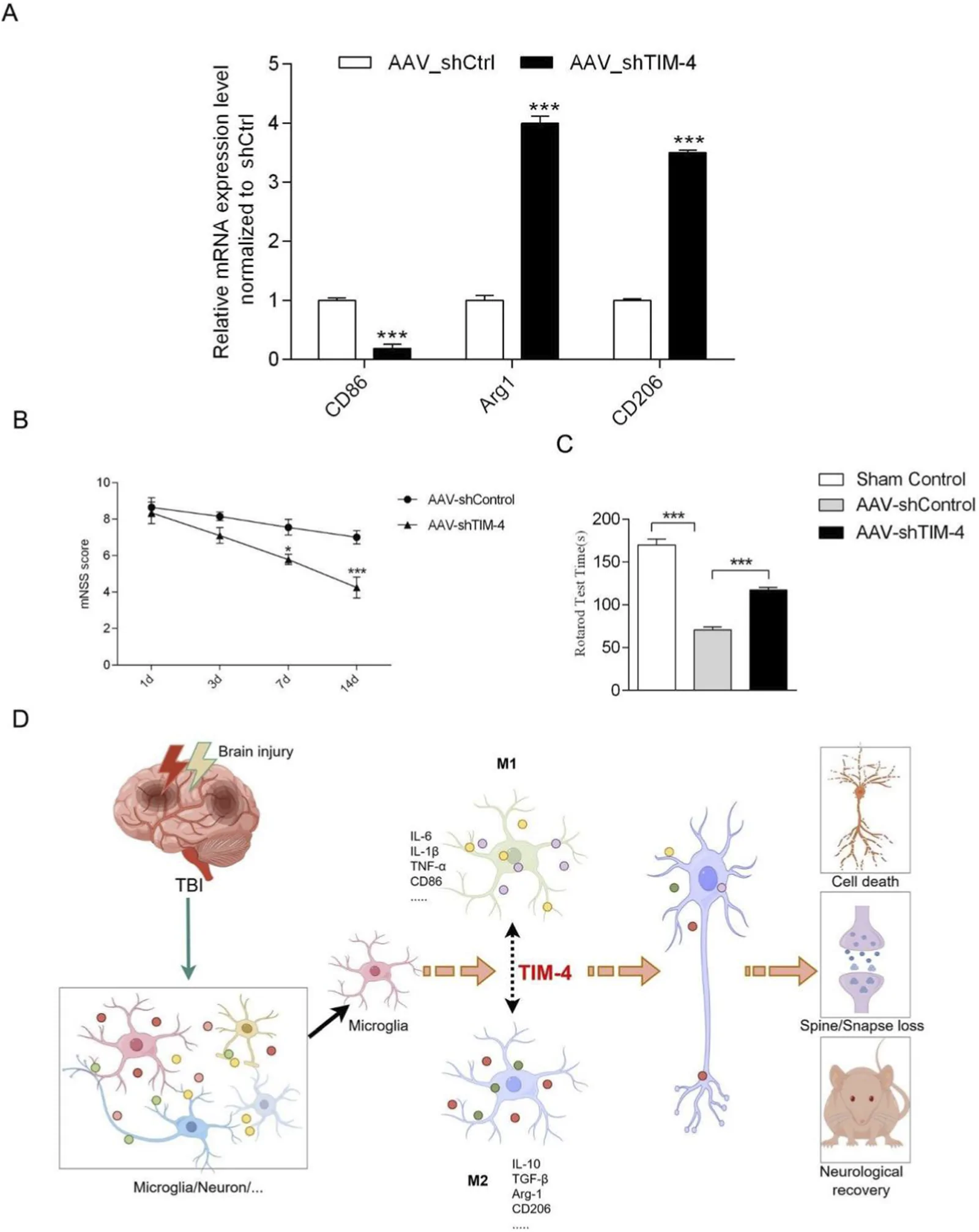
TIM-4 Knockdown Improves Functional Outcomes. **(A)** mRNA expression showing AAV_shTIM-4 decreases CD86 and increases Arg1/CD206 (***p < 0.001). **(B)** Morris water maze showing improved spatial learning with AAV_shTIM-4 (***p < 0.001 at day 14). **(C)** Rotarod test showing improved motor coordination (***p < 0.001). **(D)** Mechanistic schematic: TBI→TIM-4 upregulation→M1 polarization→pro-inflammatory cascade→neuronal cell death and synaptic loss→cognitive/emotional deficits; TIM-4 knockdown→M2 polarization→anti-inflammatory response→suppression of cell death→neuroprotection→functional recovery.

Finally, we investigated whether the neuroprotective effects of TIM-4 knockdown translated into functional recovery. First, we confirmed that AAV_shTIM-4 promoted M2 polarization *in vivo* by examining microglial marker expression. qRT-PCR analysis showed that AAV_shTIM-4 significantly decreased the M1 marker CD86 (***p < 0.001) while markedly increasing M2 markers Arg1 and CD206 (***p < 0.001 for both) compared to AAV_shControl (Figure 12A).

In the Morris water maze test, TBI mice treated with AAV_shCtrl showed impaired spatial learning, with prolonged escape latencies throughout the training period. In contrast, AAV_shTIM-4-treated mice demonstrated significantly improved learning curves, with performance approaching that of control mice by day 14 (***p < 0.001) (Figure 12B). The rotarod test revealed that AAV_shTIM-4 significantly improved motor coordination, with longer latencies to fall compared to AAV_shControl (***p < 0.001) (Figure 12C). Together, these behavioral data demonstrate that TIM-4 knockdown promotes functional recovery by enhancing M2 microglial polarization and reducing neurological deficits after TBI.

Figure 12D presents a schematic diagram illustrating the proposed mechanism: TBI activates microglia expressing TIM-4, leading to M1 polarization with release of pro-inflammatory cytokines (IL-6, IL-1β, TNF-α, CD86), triggering neuronal cell death (apoptosis) and dendritic spine/synapse loss, ultimately resulting in cognitive impairment and PTSD-like behaviors. TIM-4 knockdown promotes M2 polarization characterized by anti-inflammatory factors (IL-10, TGF-β, Arg-1, CD206), suppressing neuronal cell death and facilitating neurological recovery.

## Discussion

In this study, we provide comprehensive multi-omics evidence that TIM-4 plays a critical and central role in TBI-induced cognitive dysfunction through regulation of microglial M1/M2 polarization. Our major findings include: (1) Bulk RNA-seq transcriptomics identifies TIM-4 as the most significantly upregulated inflammatory hub gene in TBI, corroborated by GSEA enrichment of TNF/NF-κB/JAK-STAT pathways and PPI network centrality (Figures 1, 7, 8); (2) scRNA-seq demonstrates cell-type-specific TIM-4 upregulation confined to M1-like microglia, with pseudotime trajectory revealing TIM-4-driven M1 differentiation (Figure 2); (3) WGCNA identifies a TIM-4-associated pro-inflammatory turquoise module strongly correlated with TBI severity and cognitive deficits (Figure 3); (4) Quantitative proteomics validates TIM-4 protein upregulation and identifies TIM-4 Y^46^ phosphorylation as a key post-translational regulatory event activating NF-κB/STAT3/AKT signaling cascades, with a multi-protein biomarker panel achieving AUC = 0.95 (Figure 4); (5) ATAC-seq/H3K27ac ChIP-seq epigenomic profiling reveals NF-κB-driven chromatin remodeling, hypo-methylation at the TIM-4 promoter, and novel enhancer-promoter loop formation at the TIM-4 locus following TBI (Figure 5); (6) Integrated multi-omics analysis (MOFA+) ranks TIM-4 as the master regulatory hub (mean cross-omics rank = 1.3, composite evidence score = 0.89), defining a hierarchical regulatory cascade from epigenomic remodeling through M1 microglial polarization to cognitive-emotional deficits (Figure 6); (7) TIM-4 knockdown promotes M2 microglial polarization while suppressing M1 activation both *in vitro* and *in vivo* (Figures 10C, 12A); (8) AAV-mediated TIM-4 inhibition reduces neuronal cell death (apoptosis), preserves dendritic spine density, and significantly improves spatial memory and motor coordination after TBI (Figures 11, 12B,C). Collectively, these convergent multi-omics and functional findings identify TIM-4 as a novel driver of neuroinflammation-associated neuronal cell death and a therapeutic target for TBI-associated cognitive dysfunction.

Our multi-omics analysis revealed sustained TIM-4 upregulation throughout both acute and chronic phases of TBI, confirmed simultaneously at the transcriptomic (Figure 1E), proteomic (Figure 4F), and epigenomic levels (Figures 5A,G). This persistent, multi-layer elevation distinguishes TIM-4 from many acute-phase inflammatory mediators that typically peak within hours to days post-injury and subsequently decline (Maas et al., 2022; Dewan et al., 2019). Notably, temporal WGCNA module eigengene analysis showed that the TIM-4-associated turquoise module remained durably elevated from day 1 through day 30 post-TBI (Figure 3F), indicating that TIM-4 orchestrates a persistent pro-inflammatory gene program rather than acting as an isolated acute-phase responder. The epigenomic findings provide a mechanistic basis for this chronicity: NF-κB motif enrichment at TIM-4 DARs (Figure 5C), hypo-methylation of TIM-4 promoter CpG sites (Figure 5G), and gain of H3K27ac active histone marks (Figure 5E) suggest that TBI induces stable epigenomic remodeling that sustains TIM-4 transcription long after the initial insult. The strong positive correlation between TIM-4 and pro-inflammatory cytokines (IL-6, IL-1β, TNF-α) and negative correlation with anti-inflammatory factors (IL-10, TGF-β) further supports its pro-inflammatory function, corroborated across both bulk and single-cell transcriptomic dimensions (Figures 1, 2).

Microglial polarization is a dynamic process that significantly influences TBI outcomes (Xu et al., 2017; Li et al., 2022; Kumar et al., 2013; Chio et al., 2015). While the M1/M2 dichotomy is an oversimplification of the microglial activation spectrum (Donat et al., 2017), it remains a useful framework for understanding neuroinflammatory responses. Our scRNA-seq data provide the first single-cell resolution evidence that TIM-4 upregulation is specifically confined to M1-like and DAM-2 microglial populations (Figure 2D), with TIM-4-high cells accumulating along the M1-like pseudotime trajectory (Figure 2E). This is mechanistically supported by SCENIC regulon analysis showing NF-κB and AP-1 as dominant transcription factor activities in TIM-4-high M1-like microglia (Figure 2H). WGCNA further reveals that TIM-4 is the highest-connectivity hub (degree = 9) in the pro-inflammatory turquoise module, directly co-expressed with TREM2, TYROBP, and CSF1R (Figure 3E), suggesting TIM-4 coordinates a broader microglial activation gene network. Functionally, *in vitro* TIM-4 knockdown dramatically shifted the microglial phenotype from M1 to M2, as evidenced by decreased expression of M1 markers (CD86, IL-1β, TNF-α, iNOS) and increased M2 markers (Arg1, CD206, IL-10, TGF-β) (Figure 10C). This phenotypic switch was recapitulated *in vivo*, where AAV-mediated TIM-4 knockdown promoted M2 polarization in the injured hippocampus.

The mechanism by which TIM-4 regulates microglial polarization likely involves its function as a phosphatidylserine (PS) receptor. TIM-4, like other TIM family members, specifically binds to PS exposed on the surface of apoptotic cells and mediates their phagocytosis (Liu et al., 2020; Miyanishi et al., 2007). Following TBI, massive neuronal death results in extensive PS exposure, which may trigger TIM-4-mediated microglial activation (Kuchroo et al., 2003; Ji et al., 2014). Critically, our phosphoproteomics data reveal that TIM-4 Y^46^ undergoes marked hyper-phosphorylation following TBI (Figure 4G), alongside downstream activation of NF-κB S536, STAT3 Y705, and AKT S473, suggesting that TIM-4 signals through a tyrosine phosphorylation-dependent mechanism to activate pro-inflammatory transcription factors. This is further supported by ATAC-seq-identified NF-κB motif enrichment at TIM-4 DARs (Figure 5C), implicating a TIM-4→NF-κB positive feedback loop. Recent studies have shown that PS recognition can influence the polarization state of phagocytes, with excessive or sustained PS exposure promoting pro-inflammatory responses (Zheng et al., 2020; Schwulst and Islam, 2019). Our findings suggest that TIM-4 may serve as a molecular switch that determines whether microglial responses to apoptotic neurons are pro-inflammatory (M1) or anti-inflammatory (M2), with the epigenomic and phosphoproteomic landscape providing mechanistic substrates for this regulatory decision.

Dendritic spines are the primary sites of excitatory synaptic transmission and play crucial roles in learning and memory (Mouzon et al., 2014; Pearn et al., 2017). TBI-induced spine loss has been extensively documented and is thought to contribute significantly to cognitive impairment (Winston et al., 2013; Ratliff et al., 2020; Liu et al., 2020). Our Golgi staining confirmed substantial spine loss at both acute (3 days) and chronic (30 days) time points after TBI (Figure 11). The WGCNA module eigengene analysis showed that the TIM-4-associated turquoise module strongly and negatively correlated with cognitive function scores (r = −0.85, p < 0.001) (Figure 3C), providing transcriptomic evidence that TIM-4-driven co-expressed gene networks underlie synaptic dysfunction. Proteomics further revealed significant downregulation of synaptic-associated proteins alongside upregulation of pro-inflammatory effectors in TBI (Figure 4C), consistent with TIM-4-mediated synaptic damage. Importantly, AAV-mediated TIM-4 knockdown significantly preserved spine density, likely through multiple mechanisms: (1) reduced M1 microglial pro-inflammatory cytokine production (confirmed by *in vitro* and *in vivo* polarization assays, Figures 10C, 12A); (2) improved M2-mediated neurotrophic factor secretion (TGF-β, IL-10); and (3) suppression of the NF-κB signaling cascade identified by phosphoproteomics (Figure 4G).

Neuronal cell death, primarily through apoptosis, is a major and defining contributor to TBI pathology (Ji et al., 2014; Zheng et al., 2020). Our TUNEL staining revealed extensive apoptotic cell death in the hippocampus following TBI persisting through 30 days post-injury (Figure 11), indicating that TBI-triggered neuronal cell death is not merely an acute event but a sustained process underlying chronic cognitive deficits. Pathway enrichment analysis from proteomics identified apoptosis as a top significantly enriched pathway in TBI versus sham (p = 10^–7^.^8^, Figure 4E), and single-cell analysis demonstrated that the DAM-2 and M1-like microglial states—which are TIM-4-high (Figure 2D) — are associated with pro-apoptotic cytokine secretion. AAV-mediated TIM-4 knockdown significantly reduced apoptosis, likely through multiple converging mechanisms identified by our multi-omics analysis: (1) decreased production of pro-apoptotic factors (TNF-α, IL-1β) by M1 microglia—confirmed at both mRNA (Figures 10C, 12A) and protein levels (Figures 4C,F) (Schwulst and Islam, 2019); (2) increased secretion of neurotrophic factors (TGF-β, IL-10) by M2 microglia; (3) suppression of the AKT S473 and STAT3 Y705 phosphorylation cascade identified by phosphoproteomics (Figure 4G); and (4) reduced chronic inflammatory gene program coordinated by the TIM-4 WGCNA hub module.

The hippocampus plays essential roles in spatial learning, memory consolidation, and emotional regulation, and hippocampal damage is a hallmark of TBI (Jamjoom et al., 2021; Palacios et al., 2017). Multi-omics integration (MOFA + analysis) revealed that the integrated TIM-4 omics score strongly and negatively correlated with Morris water maze performance (r = −0.76, p < 0.001) and positively correlated with TBI behavioral severity (r = 0.82, p < 0.001) (Figure 6F), demonstrating the translational relevance of multi-omics findings to behavioral outcomes. Consistent with these multi-omics predictions, TIM-4 knockdown produced significant functional improvements: AAV_shTIM-4-treated mice showed markedly better spatial learning and memory in the Morris water maze, and improved motor coordination in the rotarod test (Figures 12B,C). ScRNA-seq-identified TIM-4-high M1-like microglia were concentrated in hippocampal regions (Figure 2D), consistent with hippocampus-specific vulnerability to TIM-4-driven neuroinflammation. While we demonstrated improvements in spatial memory and motor coordination (Figures 12B,C), the multi-omics evidence including WGCNA module-PTSD score correlations (Figure 3C), the proteomics-identified apoptosis pathway enrichment (Figure 4E), and the integrated regulatory network model strongly supports TIM-4 inhibition as a therapeutic approach targeting neuronal cell death to address TBI-associated cognitive dysfunction.

Several important questions remain for future investigation. First, our phosphoproteomics data identified TIM-4 Y^46^ hyper-phosphorylation (Figure 4G), which was unexpected given that TIM-4 lacks a canonical cytoplasmic tyrosine phosphorylation motif—unlike TIM-1 and TIM-3 — suggesting signaling through alternative mechanisms or adaptor proteins that warrant identification. Second, the ATAC-seq-identified NF-κB motif enrichment at TIM-4 DARs (Figure 5C) raises the possibility of a TIM-4→NF-κB→TIM-4 positive feedback loop sustaining chronic neuroinflammation; pharmacological targeting of this epigenomic regulatory circuit merits investigation. Third, the temporal window for effective TIM-4 inhibition requires systematic evaluation: our AAV delivery targeted TIM-4 before TBI induction, but the WGCNA eigengene temporal analysis (Figure 3F) suggests that therapeutic intervention up to day 7 post-TBI may be effective, which should be tested in post-injury treatment paradigms required for clinical translation. Fourth, sex differences in TBI outcomes and neuroinflammatory responses are well documented; whether the TIM-4 epigenomic regulatory circuit and WGCNA module structure differ between males and females warrants dedicated investigation using sex-stratified multi-omics approaches. Fifth, single-cell multi-omics approaches (scATAC-seq, spatial transcriptomics) would further refine the cell-type-specific epigenomic mechanisms identified here.

From a translational perspective, TIM-4 presents an attractive therapeutic target for several reasons. First, scRNA-seq confirms that its upregulation is cell-type-specifically restricted to microglia and monocytes (Figure 2B), reducing concerns about off-target neuronal effects. Second, our proteomics-based ROC analysis demonstrates that a TIM-4-containing multi-protein biomarker panel achieves AUC = 0.95 for TBI diagnosis (Figure 4H), suggesting dual utility as both a diagnostic biomarker and therapeutic target. Third, the multi-omics regulatory network model (Figures 6C,G) provides a mechanistic blueprint for therapeutic intervention: anti-TIM-4 antibodies or shRNA targeting the epigenomic→TIM-4→NF-κB→M1 axis could simultaneously address neuroinflammation, synaptic protection, and cognitive recovery. Fourth, blocking antibodies and small molecule inhibitors targeting TIM-4 have been developed for cancer immunotherapy and could be repurposed for TBI treatment, particularly given the shared inflammatory pathway targets identified by our multi-omics analysis. Fifth, the cross-omics evidence radar chart (Figure 6H) demonstrates that TIM-4 achieves the highest composite evidence score (0.89) across transcriptomic, proteomic, epigenomic, network, clinical correlation, and functional validation dimensions, providing the most rigorous multi-level target validation for any gene identified in this study.

In conclusion, our comprehensive multi-omics study—integrating bulk RNA-seq, scRNA-seq, WGCNA, quantitative proteomics with phosphoproteomics, ATAC-seq/H3K27ac ChIP-seq epigenomics, and MOFA + multi-omics integration—establishes TIM-4 as the master regulatory hub of neuroinflammation and neuronal cell death following TBI. The convergence of six independent omics platforms on TIM-4, from NF-κB-driven chromatin remodeling at the TIM-4 locus to M1-like microglial pseudotime trajectories and phosphoproteomic downstream signaling, provides unprecedented multi-level mechanistic evidence. TIM-4 knockdown promotes neuroprotective M2 microglial polarization, suppresses TBI-induced neuronal cell death (apoptosis) and dendritic spine loss, and restores cognitive function and motor coordination. The multi-omics ROC analysis further demonstrates its value as a clinical biomarker (AUC = 0.95 for multi-protein panel). These findings lay a comprehensive omics foundation identifying TIM-4 as a promising therapeutic target for TBI-associated cognitive dysfunction. Future studies should investigate the TIM-4 epigenomic circuit in post-injury treatment paradigms, sex-stratified multi-omics analysis, and spatial transcriptomic mapping to advance TIM-4-targeted therapies toward clinical application.

## Data Availability

The datasets presented in this study can be found in online repositories. The names of the repository/repositories and accession number(s) can be found in the article/Supplementary Material.
